# Molecular and Physiological Study of *Candida albicans* by Quantitative Proteome Analysis

**DOI:** 10.3390/proteomes6030034

**Published:** 2018-09-18

**Authors:** Seiji Shibasaki, Miki Karasaki, Wataru Aoki, Mitsuyoshi Ueda

**Affiliations:** 1 General Education Center, Hyogo University of Health Sciences, 1-3-6 Minatojima, Chuo-ku, Kobe, Hyogo 650-8530, Japan; seiji@huhs.ac.jp (S.S.); m.karasaki@huhs.ac.jp (M.K.); 2 Graduate School of Agriculture, Kyoto University, Kyoto 606-8502, Japan; aoki.wataru.6a@kyoto-u.ac.jp

**Keywords:** *Candida albicans*, macrophage, monolithic silica capillary column, vaccine, virulence

## Abstract

*Candida albicans* is one of the major pathogens that cause the serious infectious condition known as candidiasis. *C. albicans* was investigated by proteome analysis to systematically examine its virulence factors and to promote the development of novel pharmaceuticals against candidiasis. Here, we review quantitative time-course proteomics data related to *C. albicans* adaptation to fetal bovine serum, which were obtained using a nano-liquid chromatography/tandem mass spectrometry system equipped with a long monolithic silica capillary column. It was revealed that *C. albicans* induced proteins involved in iron acquisition, detoxification of oxidative species, energy production, and pleiotropic stress tolerance. Native interactions of *C. albicans* with macrophages were also investigated with the same proteome-analysis system. Simultaneous analysis of *C. albicans* and macrophages without isolating individual living cells revealed an attractive strategy for studying the survival of *C. albicans*. Although those data were obtained by performing proteome analyses, the molecular physiology of *C. albicans* is discussed and trials related to pharmaceutical applications are also examined.

## 1. Introduction

*Candida albicans* is a commensal yeast of humans that is found in the microflora of the oral cavity, skin, gastrointestinal tract, and vagina [1,2,3]. *C. albicans* can cause opportunistic systemic or superficial candidiasis when the host immunity is compromised by cancer chemotherapies, administration of immunosuppressants, or acquired immune deficiency syndrome (AIDS). The mortality rate of systemic candidiasis is approximately 50% because few effective pharmacotherapeutic options or diagnostic methods exist [4]. Attaining a comprehensive understanding of the molecular and physiological aspects of *C. albicans* is key for developing novel drugs.

In recent years, proteome analysis progressed as an effective approach for characterizing dynamic variations of biological systems. *C. albicans* was also studied using proteomics to measure changes during adaptation to a variety of conditions. Typically, those proteome changes were characterized by endpoint analyses based on two-dimensional polyacrylamide gel electrophoresis (2D-PAGE) [5,6,7]. Those reports showed many valuable findings depicting reference maps of two different conditions. However, 2D-PAGE has several problems including limitations in terms of separation factors, molecular masses, and isoelectric points. Furthermore, the low-throughput aspect of 2D-PAGE is disadvantageous in time-course analysis studies involving numerous samples from target cells. The shotgun approach is recognized as an alternative method of proteome analysis. The shotgun approach consists of liquid chromatography and tandem mass spectrometry (LC–MS/MS), and enables identification of many proteins in a high-throughput manner, including low-abundance proteins. Additionally, a system developed with ultra-performance in terms of chromatographic separation showed excellent performance [8]. Monolithic silica provides highly efficient separation as a long column because of its high permeability [9], and it was applied for separating tryptic fragments in mixed samples with a shallow gradient. For instance, an LC–MS/MS system with a long monolithic silica capillary column (500 cm) was used to identify approximately 2600 proteins produced in the human arthritic cell line MH7A in a single run [10]. Based on these advantages, proteome analyses of *C. albicans* progressed in recent years. Here, we mention the molecular and physiological characteristics of *C. albicans* virulence and pharmaceutical applications using proteome data.

## 2. Time-Course Proteomics Analysis of *C. albicans* Adaptation to Serum

*C. albicans* virulence is based on the secreted aspartic protease family [11,12,13], the yeast-to-hyphal transition [14,15], and the agglutinin-like sequence family [16,17,18]. Utilizing these molecules or functions, *C. albicans* adheres to human tissues, invades epithelial cells, and disseminates into the bloodstream. Although serum adaptation is an indispensable function for *C. albicans*, the factors described above do not clearly account for the overall adaptation mechanism. Thus, time-course proteome analyses of *C. albicans* adaptation to fetal bovine serum (FBS) was investigated using LC–MS/MS and a long monolithic silica capillary column [19].

With the aim of focusing on early-stage adaptation to serum, the *C. albicans* strain SC5341 was first grown in yeast extract, peptone, dextrose (YPD) media and then transferred to yeast nitrogen base (YNB) media containing 10% (*v*/*v*) FBS (YNB + FBS), or YNB without FBS (YNB − FBS) as a control. Incubating *C. albicans* in YNB ± FBS is a simple model for studying early systemic candidiasis. *C. albicans* cells were recovered at sequential timepoints (0, 10, 20, 40, and 60 min). Cell morphology was determined by phase-contrast microscopy to confirm the serum-dependent hyphal extension [20]. As a result, it was confirmed that *C. albicans* maintained its yeast form at 0–10 min and started hyphal development at 20 min. The average hyphal lengths were 0, 0.06, 0.61, 3.22, or 7.23 mm at 0, 10, 20, 40, or 60 min, respectively. In another study, where *C. albicans* was in contact with Caco-2 cells for 120 min, the hyphae extensions were longer (36.9 mm) [21]. Therefore, incubating *C. albicans* in YNB + FBS for 60 min was an appropriate model for studying proteome dynamics during early-stage serum adaptation.

**Figure 1:**
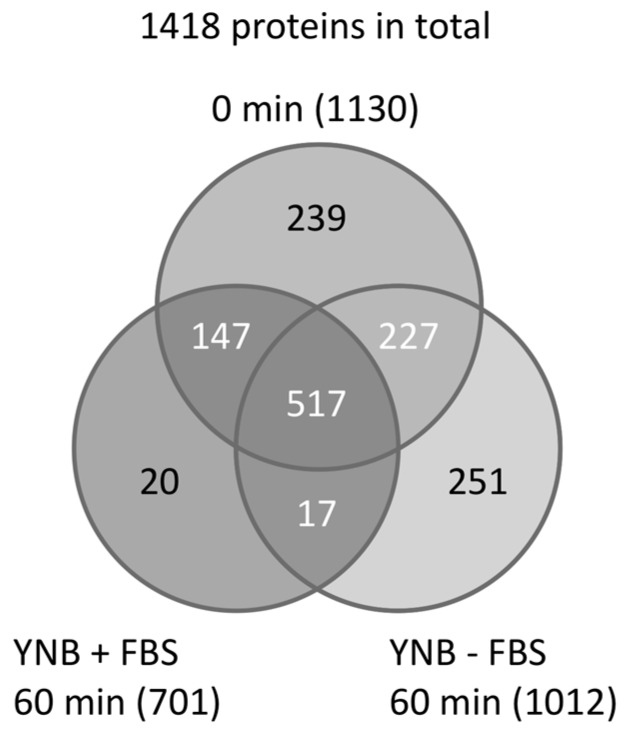
Venn diagram of proteins identified in samples at 0 and 60 min grown in yeast nitrogen base (YNB) medium ± fetal bovine serum (FBS). A total of 1418 unique proteins were identified.

Next, peptides prepared from *C. albicans* grown in YNB ± FBS (YNB with or without FBS) were subjected to LC–MS/MS analysis using a monolithic silica capillary column (200 cm) [22]. A total of 1418 unique proteins were identified, including 1130, 1012, and 701 proteins from the 0- and 60-min YNB − FBS cultures, and the 60-min YNB + FBS culture, respectively (Figure 1) [19]. Additionally, between 868 and 1034 proteins were identified from the YNB + FBS samples taken at 10, 20, and 40 min after adding FBS.

Proteins that (i) were not detected in the 60-min YNB − FBS or 0-min control samples, and that (ii) were continuously expressed (once identified) until 60 min in the YNB + FBS samples were defined as “newly produced proteins”. The newly produced proteins were regarded as major effectors that positively contributed to the cell integrity by their presence in the serum. Four proteins (ATP16, RHR2, HGT1, and orf19.3767) were first identified after a 10-min FBS exposure, and these were continuously detected at the later time points. Three of them, HGT1, orf19.3767, and ATP16, are known as transport-related molecules involved in the acquisition of glucose and ATP. *C. albicans* might prioritize the acquisition of essential elements after 10 min during the adaptation process. Indeed, HGT1, a high-affinity glucose transporter, was previously reported to be an essential molecule [23,24].

**Figure 2:**
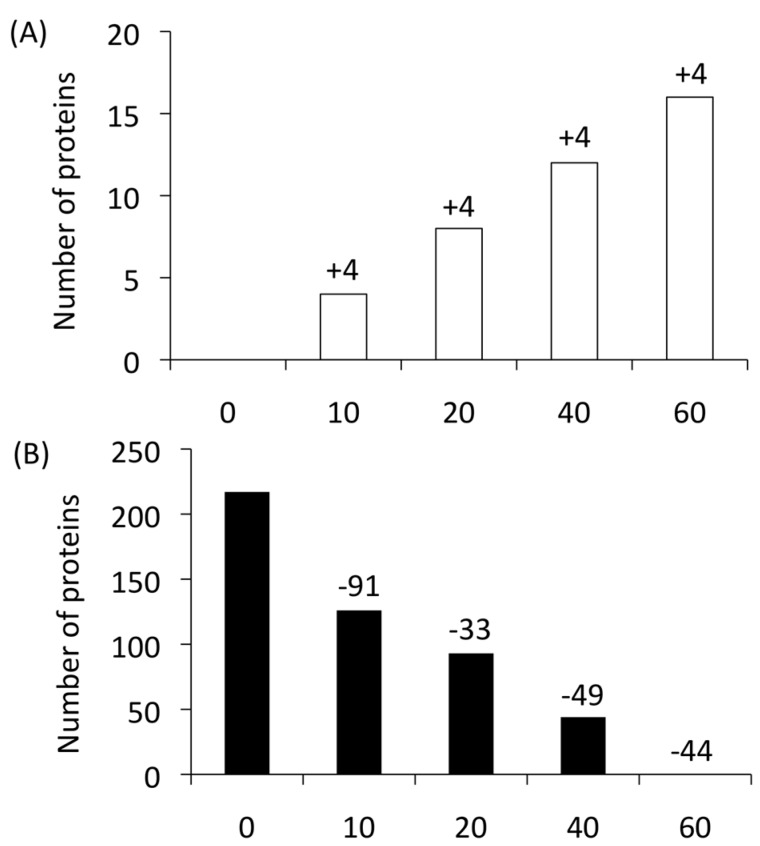
Summary of proteins identified in time-course proteome analyses of *Candida albicans*. (**A**) A total of 16 newly produced proteins during serum adaptation were identified. (**B**) The number of disappearing proteins.

Four newly produced proteins were each found at 20, 40, and 60 min (Figure 2A) [19]. Thus, 16 proteins total (ATP16, RHR2, HGT1, SPT14, ERG6, PEX12, orf19.3767, orf19.713, orf19.3686, orf19.4825, orf19.4594, orf19.4620, orf19.5342.2, orf19.2439, orf19.4123, and orf19.6211) were categorized as newly produced proteins.

Conversely, proteins that (i) were continuously detected from 0 min to a subsequent time point, that (ii) were not detected at any time point after their expression first disappeared, and that (iii) were not detected in the 60 min YNB − FBS sample were defined as “disappearing proteins”. In total, 217 proteins were identified as disappearing proteins (Figure 2B). These proteins are thought to provide advantages under nutrient-rich conditions, whereas they may have disadvantages in a severe environment or may be unnecessary.

## 3. Quantitative Time-Course Proteomics Analysis of *C. albicans* Serum Adaptation

Previous investigators also conducted a quantitative time-course proteomics study of *C. albicans* during the early stages of serum adaptation, from 0–180 min [25]. Quantitative time-course proteome analysis requires a high-throughput method when measuring numerous samples. In that study [25], an LC–MS/MS system was equipped with a monolithic silica capillary column longer (470 cm) than that described in the previous section [19]. Comprehensive characterization of the adaptation process using quantitative time-course proteome analysis is expected to enhance the understanding of *C. albicans* virulence. Previously uncharacterized *C. albicans* proteins were identified as possible virulence factors.

**Figure 3:**
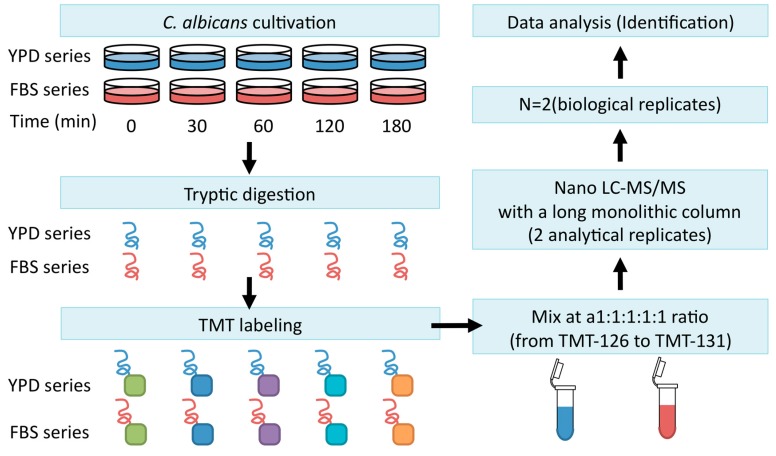
The workflow for identifying proteins. *C. albicans* was cultured in yeast extract, peptone, dextrose (YPD) or FBS medium for 0, 30, 60, 120, or 180 min. Cells were disrupted and lysates were digested with trypsin. Tryptic peptides were labeled with tandem mass tagging (TMT) and subjected to LC–MS/MS analysis with a long monolithic column.

Firstly, *C. albicans* strain SC5314 was incubated for 2 h at 37 °C to maintain it in exponential growth phase, after which the cells were harvested and transferred to YPD medium (YPD series) or YPD + FBS (FBS series). Extracted cellular proteins were labeled using tandem mass tagging (TMT). Continuous LC–MS/MS analysis was conducted with a long monolithic silica capillary column (470 cm) (Figure 3) [25].

**Figure 4:**
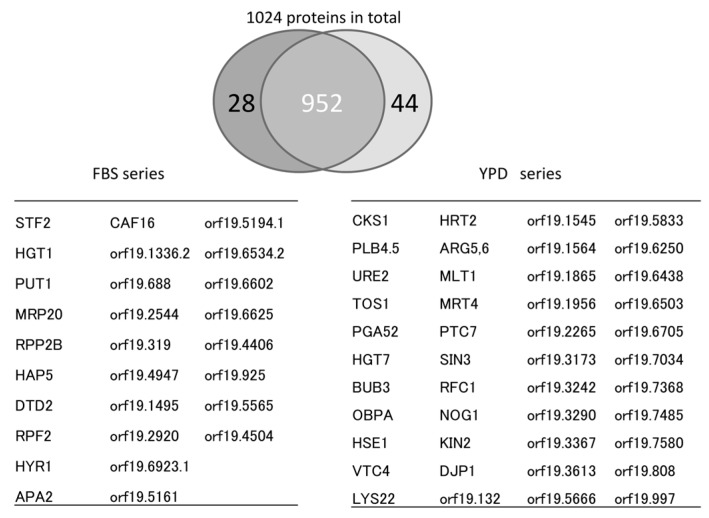
Summary of proteins identified in YPD- or FBS-specific conditions. A total of 1024 proteins were identified. The number of proteins common to both the YPD and FBS series was 952.

Proteome analyses were conducted using an LC (Ultimate 3000)/MS (LTQ Velos Orbitrap mass spectrometer, Waltham, MA, USA) system. The system separated prepared tryptic digests at a flow rate of 500 nL·min^−1^. The mass spectrometry data were used for identification, and quantification was performed using the Proteome Discoverer 1.2 software. Protein identification was performed using MASCOT against the Assembly 21 protein database in the *Candida* genome database (CGD) [26]. As a result, 1024 proteins were identified and quantified. Of these proteins, 44 were categorized as YPD-specific and 28 were categorized as FBS-specific (Figure 4).

**Figure 5:**
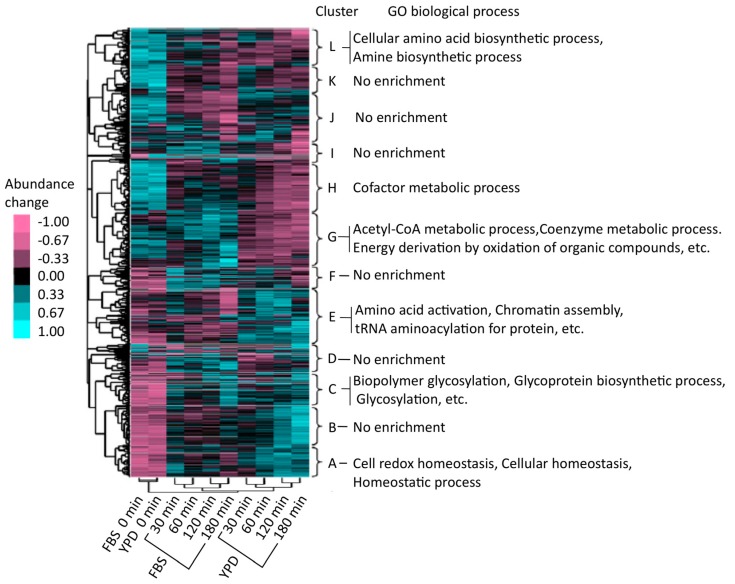
Hierarchical clustering analysis. The mean value of each row (i.e., each protein) was set at 0, and the sum of the squares of the values of each row was 1.0. Color bars indicate changes in protein abundance. Up- and downregulated proteins are shown in cyan and magenta, respectively.

The Cluster 3.0 software [27] was used for hierarchical cluster analysis of the regulatory patterns of protein abundance. Proteins were hierarchically clustered (on the vertical axis) and associated with 12 characteristic categories, labeled A to L (Figure 5) [25]. Four types of groups were identified, i.e., groups with an increasing trend both in the YPD and FBS series (A–D), a cluster that showed increased expression in the YPD series (E), groups that showed increased expression in the FBS series (F and G), and other groups (H–L). To functionally categorize these groups, the proteins were examined by gene ontology (GO) enrichment analysis using the Database for Annotation, Visualization, and Integrated Discovery (DAVID) (http://david.abcc.ncifcrf.gov/) [28]. As a result, it was found that groups A–D were enriched in proteins related to cellular homeostasis, redox regulation, and glycoprotein metabolism. Proteins in cluster E (YPD-specific) were associated with aminoacyl transfer RNA (tRNA) biosynthesis. Cluster G (FBS-specific) was enriched with proteins involved in intracellular processes such as catabolic acetyl coenzyme A (CoA) catabolism and coenzyme catabolic processes related to the tricarboxylic acid (TCA) cycle (also known as the citrate cycle). Proteins in the TCA cycle were upregulated in the FBS series compared to those in the YPD series; many proteins involved in the TCA cycle (for example, Aco1, Aco2, Idp1, Idp2, Fum12, Kgd1, Mdh1, Pck1, and Sdh12) were enriched in cluster G. In human blood, *C. albicans* might optimize its proteome by upregulating the TCA cycle to efficiently acquire energy. This observation was in accord with an earlier study using a microarray that showed that human blood and a polymorphonuclear cell fraction could transcriptionally activate the TCA cycle [29,30]. Furthermore, other investigations demonstrated that Gcn4 [31,32], a transcriptional activator, was important in upregulating the TCA cycle [33,34].

**Figure 6:**
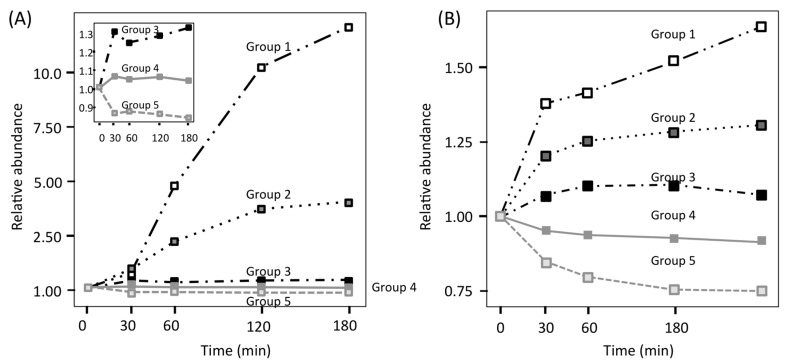
Grouping of individual protein profiles by *k*-means clustering. (**A**) FBS series; (**B**) YPD series. For the FBS series, the detailed profiles of groups 3–5 are depicted in the magnified panel.

To investigate treatment-specific proteome patterns, time-course profiles of the FBS and YPD series were categorized using non-hierarchical *k*-means clustering. Two protein clusters with considerable upregulation in the FBS series (group 1: tenfold, group 2: fivefold) were confirmed (Figure 6A) [25], whereas proteins in the YPD series only showed slow changes, with a maximum upregulation of approximately twofold (Figure 6B) [25]. In the FBS series, only two (Sod5 and Blp1) of the 1024 identified proteins were clustered in group 1, and four proteins (Ece1, Hgt1, Stf2 and Ucf1) were clustered in group 2. Other proteins in the FBS series showed slow changes in abundance (approximately twofold). Based on these results, it was suggested that *C. albicans* employed the following adaptation strategy: firstly, *C. albicans* tuned its proteome to adapt to a new environment, in which several proteins were upregulated twofold more than suggested by previous reports [5,35,36]. Secondly, a few proteins were upregulated by over fivefold or 1tenfold, which might suggest that these proteins are important for adaptation to the different environment. These findings conflict with some data presented in earlier transcriptome reports, which showed that dozens of proteins were upregulated by over fivefold after blood treatment [29,30], indicative of a low correlation between transcriptome and proteome analyses of *C. albicans*, owing to differences in the stability of transcripts and proteins. A low correlation between transcriptome and proteome was also reported by Edfors et al. [37]. Use of the RNA-to-protein conversion factor is suggested to normalize their correlation.

Proteins uniquely identified or specifically upregulated in the FBS series can potentially take part in serum adaptation. Twenty-two proteins were found that were specifically upregulated in the FBS series [25]. In addition, 28 proteins were uniquely identified in the FBS series. These 50 proteins were designated “FBS-induced proteins”. Several previously reported virulence factors (for example, Alo1, Nag6, Phr1, Rpf2, and Sod5 [38,39,40,41,42]), were included in this group, indicating that these proteins found by proteome analysis are potential virulence factors.

**Figure 7:**
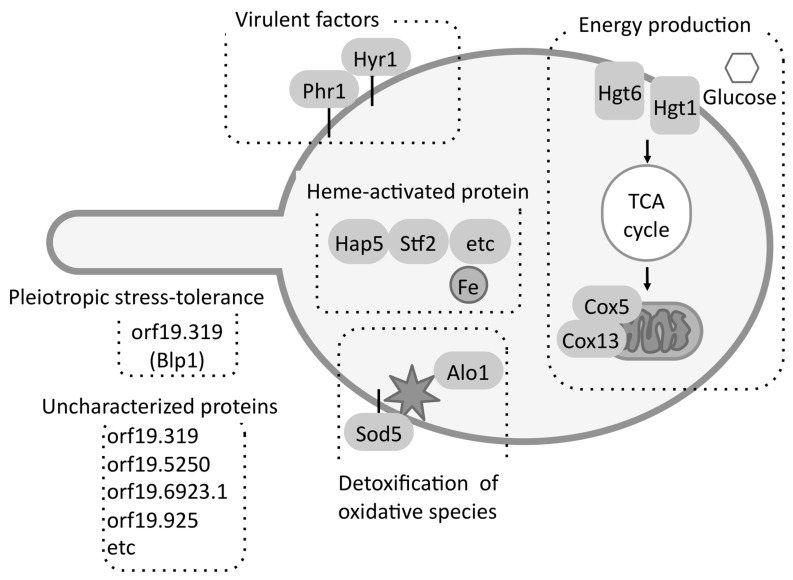
The process of adaptation to FBS. *C. albicans* induced the expression of proteins related to energy production, the elimination of oxidative species, iron acquisition, virulence, pleiotropic stress tolerance, and uncharacterized processes.

Several proteins among the 50 FBS-induced proteins were related to detoxification of oxidative species, high-affinity glucose transport, the TCA cycle, oxidative phosphorylation, and iron uptake (Figure 7) [25]. Recently, Ahmed et al. [43] also suggested that, in addition to rhw TCA cycle, amino-acid and fatty-acid metabolism were upregulated under FBS-induced condition. In addition, a possible virulence factor orf19.4914.1 (Blp1) showing pleiotropic stress-tolerance in *Saccharomyces cerevisiae* was identified.

## 4. Finding an Antigen for a Potential Vaccine

Constitutively expressed *C. albicans* associated with the cell wall or an important metabolic pathway are thought to be suitable antigen candidates for producing a vaccine. In an earlier proteomics study of hyphal induction in *C. albicans*, several cell-wall proteins were proposed as candidate vaccine antigens [44]. The *C. albicans* malate dehydrogenase enzyme (Mdh1p, EC1.1.1.37) [45] was identified in the time-course proteome studies discussed above (group 4, Figure 6). Mdh1p is essential for completing the TCA cycle. This protein was regarded as a candidate vaccine antigen against candidiasis because it was detected at all time points studied without large variations in its relative abundance. Previously, Mdh1p was also identified in a proteome analysis using a two-dimensional gel electrophoresis/MS system [46] to screen for immunogenic *C. albicans* proteins.

Based on these circumstances, a His-tagged Mdh1p variant was initially produced in *Escherichia coli* and investigated for its immunogenicity as a candidate vaccine antigen against candidiasis [47]. Next, Mdh1p was purified using an endotoxin column and administered to mice via subcutaneous injection or intranasal administration before they were given a lethal dose of *C. albicans*. After vaccination, immunoglobulin G (IgG) antibody responses were evaluated by performing enzyme-linked immunosorbent assays (ELISAs). Furthermore, survival tests were performed to evaluate the efficacy of *C. albicans* Mdh1p as a vaccine.

**Figure 8:**
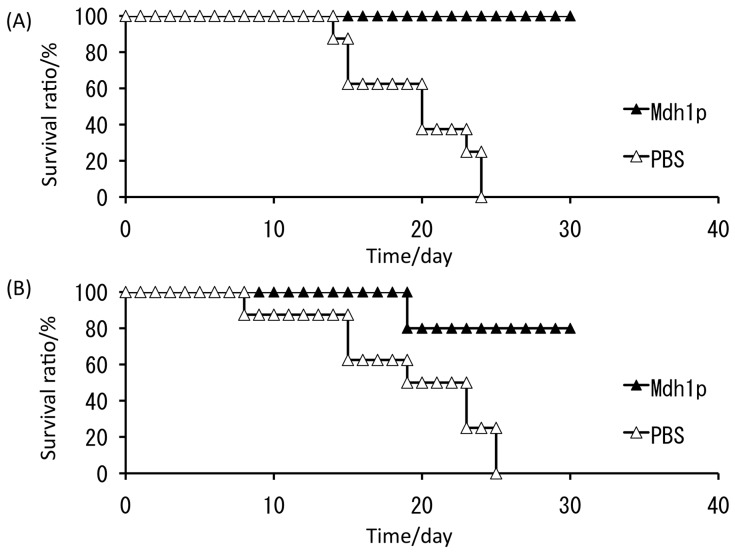
Survival ratio after challenging of lethal dose of *C. albicans*. The antigen, malate dehydrogenase enzyme (Mdh1p), was administered with cholera toxin as an adjuvant to mice prior to the *C. albicans* challenging. (**A**) Subcutaneous injection of Mdh1p; (**B**) intranasal administration of Mdh1p. Triangles, administration of phosphate-buffered saline (PBS); closed triangles, administration of Mdh1p. Vaccinated mice infected with a lethal dose of *C. albicans* (at day 0) had a significantly prolonged survival time compared with mice administered the control (*p* < 0.01).

All control mice died within 25 days, whereas 100% and 80% of mice treated with subcutaneous and intranasal administration of Mdh1p, respectively, survived (Figure 8) [47]. This investigation suggested that, among the *C. albicans* antigens examined thus far, such as hyphal wall protein (Hwp1p) [48], phosphoglycerate kinase (Pgk1p) [49], and glyceraldehyde-3-phosphate dehydrogenase (Gap1p) [50], Mdh1p is currently the most effective antigen for use as a vaccine for *C. albicans*. Further studies of time-course variation in *C. albicans* under serum-containing conditions to identify virulence-related molecules would also provide other effective antigenic proteins. Those potential antigens should be presented to the host using an effective tool, such as a molecular display system [51,52]. Presently, we can choose different types of display systems for producing oral vaccines with potential antigens [53,54].

## 5. Mixed and Quantitative Proteome Analysis

**Figure 9:**
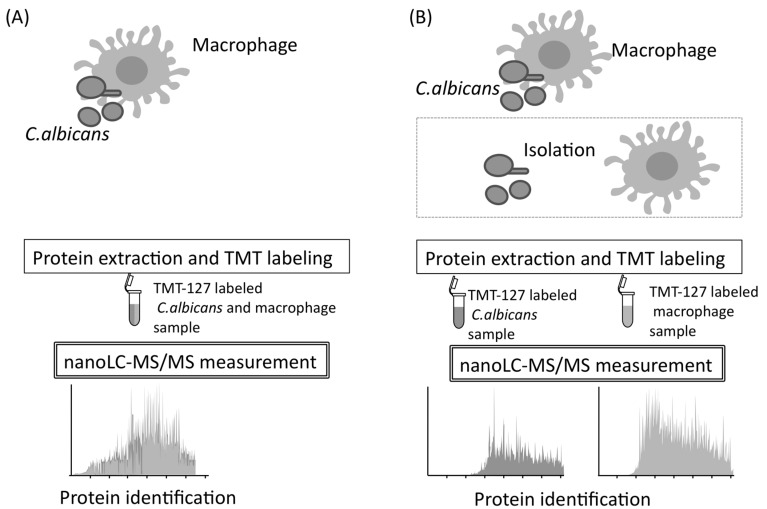
The process of mixed and quantitative proteome analysis. (**A**) Protein identification is performed with *C. albicans* and *Mus musculus* genome databases without isolation of two organisms. (**B**) The same identification is performed after isolation of two organisms.

Immunological protection of the host against *C. albicans* is based on, at first, internalization of this pathogen by macrophages [55]. Macrophages can destroy microorganisms by phagocytosis and recruit several immune cells by cytokine signaling [56,57]. Unfortunately, following phagocytosis, *C. albicans* kills macrophages and eventually escapes from them [58,59]. Little is known about the mechanisms used by *C. albicans* to escape from macrophages. Therefore, mixed and quantitative proteome analysis may be useful, and it is performed to understand comprehensive proteome responses occurring during natural interactions between *C. albicans* and macrophages. To conduct mixed and quantitative proteome analysis, samples prepared from *C. albicans* and macrophages were directly analyzed by nano-LC–MS/MS without isolating the *C. albicans* and macrophage cells during co-cultivation (Figure 9) [60].

The measurement accuracy of mixed and quantitative proteome analysis was first evaluated. The standard sample was separated into three aliquots at a 0.5:1:2 ratio by volume. After labeling with TMT reagents with different reporters, the three samples were mixed in a single tube and injected into a nano-LC–MS/MS system. Each peptide showed the approximate expected proportional intensity of reporter ions based on the ratio of 0.5:1:2. This experiment suggested that each peptide could be quantified at high accuracy, even if the peptides were in a mixture derived from two different cell types. Next, the amount of tumor necrosis factor (TNF)-α released from macrophages into the culture medium was investigated by ELISA, because macrophages infected by pathogens produce TNF-α [61,62]. The amount of TNF-α produced from macrophages interacting with *C. albicans* was greater than non-interacting controls and increased in a time-dependent manner, with the amount of TNF-α increasing after 3 h of interaction. To identify proteins related to the mechanism whereby *C. albicans* escapes from macrophages, an early time point (3 h) was selected for the proteome analysis.

After protein isolation, the investigators performed reduction, alkylation, digestion, TMT-labeling, and LC–MS/MS measurements, using the same system described in the above section. The MS data for each biological replicate was used for protein identification and quantification. Protein identification was performed using MASCOT against the Assembly 21 CGD for *C. albicans* and against the *Mus musculus* database in the National Center for Biotechnological Information (http://www.ncbi.nlm.nih.gov/) and the International Protein Index. As a result, 483 *C. albicans* proteins and 1253 macrophage proteins were identified by performing mixed and quantitative proteome analysis (Figure 9). Using the *C. albicans* database, 976, 18, and 0 proteins were identified from the *C. albicans* monoculture, the macrophage monoculture, and complete culture medium used as background, respectively.

Apparently up- and downregulated *C. albicans* proteins were categorized based on their functions by pathway analysis using the KEGG pathway of DAVID (threshold: enrichment score > 1.5) [60]. Ninety-five of the upregulated proteins were mainly involved in pathways associated with glucose synthesis, amino-acid degradation, proteasome functions, and stress responses. The 132 downregulated proteins were categorized mainly in the “ribosome”. Three conclusions were suggested from this pathway analysis: (1) with respect to central metabolic pathways, *C. albicans* degrades proteins through proteasomes and generates glucose from the degradation products to prevent glucose starvation; (2) *C. albicans* produces stress-tolerance proteins that help it survive inside macrophages; (3) *C. albicans* produces candidate pathogenic proteins that facilitate escape from macrophages.

**Figure 10:**
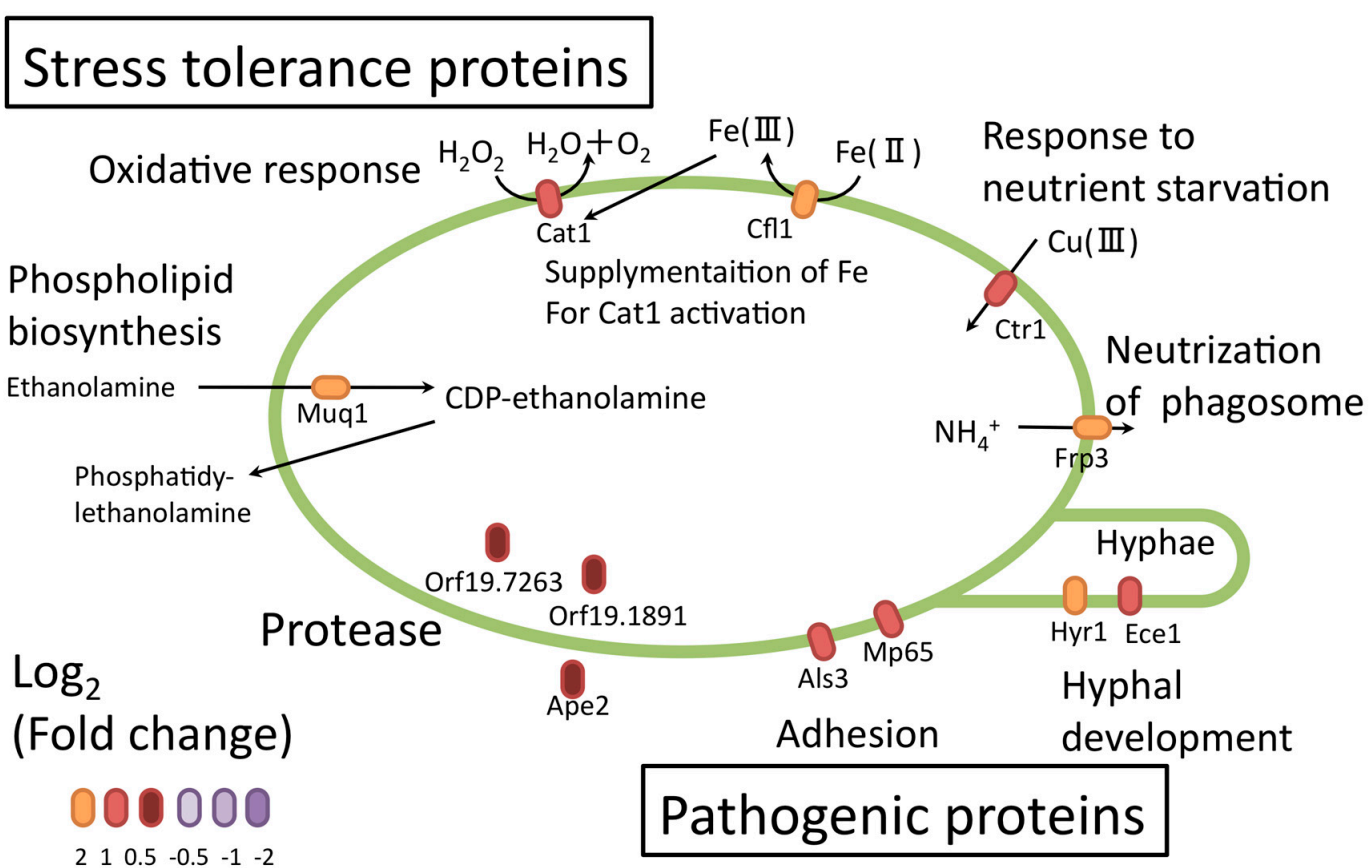
Proteins related to stress tolerance and candidate pathogenic proteins in *C. albicans*. Orange and purple ellipses indicate the fold-changes of the individual proteins.

Several upregulated proteins identified in the investigation serve roles in adhesion (Als3, Mp65) (Figure 10) [60]. Als3 promotes *C. albicans* invasion into endothelial cells by binding to cadherin and promoting its own endocytosis [63,64]. The *C. albicans* adhesion protein might further help with adhesion and escape from macrophages. Upregulation of some proteases (Ape2 [65], orf19.1891, and orf19.7263) suggested that proteolysis and peptide utilization were necessary for *C. albicans* survival. Some upregulated proteins (orf19.4914.1, orf19.4441, orf19.5201.1, orf19.6035, orf19.357, orf19.3053, and orf19.5078) related to unknown proteins or hyphal formation were not functionally characterized in detail. These proteins could be important virulent factors, and further studies could provide important insights.

The macrophage proteins whose levels changed during the interaction with *C. albicans* were also confirmed. Most of the dysregulated proteins were downregulated, not upregulated. In particular, downregulation of macrophage apoptosis-associated protein, nitric-oxide-associated protein 1 (NOA1), syntheses [66,67,68,69] and chaperone HSPA1A syntheses [70,71] suggested that *C. albicans* could evade macrophages, in part, by inhibiting the production of these macrophage proteins. These results found in the mixed and quantitative proteome provide novel insights into the relationship between *C. albicans* and macrophages, and should lead to a better understanding of systemic candidiasis and the development of novel pharmaceutical inhibitors of candidiasis.

## 6. Conclusions

In this review, quantitative proteomic studies of the virulent microorganism *C. albicans* were described. All quantitative proteome analyses described here were conducted using an LC–MS/MS system with a long monolithic silica capillary column. As an application of proteomics studies, the *C. albicans* antigen used for vaccine development was investigated here. The nano-LC–MS/MS system could contribute to understanding the physiology of *C. albicans*, as well as diagnostic or therapeutic drug development for candidiasis [72,73].
